# Analysis of an IncR Plasmid Carrying *bla*_NDM-1_ Linked to an Azithromycin Resistance Region in Enterobacter hormaechei Involved in an Outbreak in Quebec

**DOI:** 10.1128/spectrum.01998-21

**Published:** 2021-12-22

**Authors:** Florence Doualla-Bell, David A. Boyd, Patrice Savard, Khadidja Yousfi, Isabelle Bernaquez, Simon Wong, Valentine Usongo, Laura F. Mataseje, Michael R. Mulvey, Sadjia Bekal

**Affiliations:** a Laboratoire de santé publique du Québec, Institut national de santé publique du Québec, Sainte-Anne-de-Bellevue, Quebec, Canada; b National Microbiology Laboratory, Public Health Agency of Canada, Winnipeg, Manitoba, Canada; c Département de microbiologie, infectiologie et immunologie, Université de Montréal, Montréal, Quebec, Canada; d Département clinique de médecine des laboratoires OPTILAB-CHUM and CRCHUM, Université de Montréal, Montréal, Quebec, Canada; Emory University School of Medicine

**Keywords:** complex class I integron, *bla*_NDM-1_, IS*26*-*mph*(A) unit, *Enterobacter hormaechei*, IncR plasmid, multidrug resistance

## Abstract

In the context of a recent rise in prevalence of NDM-encoding carbapenemase-producing Enterobacterales (CPE) in the province of QC, Canada, the genetic environment of *bla*_NDM-1_ was investigated. Three NDM-producing clinical isolates of Enterobacter hormaechei recovered from hospitalized patients involved in a putative outbreak were further characterized by whole-genome sequencing (WGS). Two isolates were confirmed by pulsed-field gel electrophoresis and WGS to be closely related. In addition to a ∼128 kb IncFII conjugative multidrug-resistance (MDR) plasmid, these isolates possessed a ∼45 kb mobilizable IncR MDR plasmid containing 2 MDR regions: a complex class 1 integron harboring *bla*_NDM-1_ and 7 other AMR genes, and the *IS26-mph*(A)*-mrx-mphR*(A)*-IS6100* azithromycin resistance unit. The predicted antimicrobial resistance (AMR) genes correlated with the antimicrobial susceptibility testing results. The multidrug-resistant phenotype in addition to the presence of two important mobile genetic elements, suggest a potent role as a reservoir of antibiotic resistance for such a small IncR plasmid.

**IMPORTANCE** Analyzing the genetic environment of clinically relevant MDR genes can provide information on the way in which such genes are maintained and disseminated. Understanding this phenomenon is of interest for clinicians as it can also provide insight on where these genes might have been sourced, possibly supporting outbreak investigations.

Carbapenem resistance in human pathogens is a growing clinical and public health concern. This class of antimicrobials are used to treat human infections caused by multidrug-resistant (MDR) Enterobacterales. Owing to their importance in human medicine, the World Health Organization (WHO) has classified them as “High Priority Critically Important Antimicrobials” (1). The surveillance of antimicrobial resistance is critical to public health as it provides timely feedback of data to stakeholders with the goal to generate action aimed at reducing or preventing the public health threat being monitored. Not only are antimicrobial surveillance activities of national importance but these activities form part of the international response to the global threat posed by antibiotic resistance (2).

In Quebec, the carbapenemase-producing Enterobacterales (CPE) surveillance program was instituted in 2010. Over the years, we observed a gradual increase in the number of CPE reported cases of New Delhi metallo-β-lactamase (NDM) producing strains. However, this number doubled in 2019 reaching up to 20% of all Enterobacterales (3) and then attained 26% in 2020 (Quebec CPE Surveillance Program, personal communication). Plasmids are the most important drivers of carbapenemase gene dissemination, where the majority have been shown to belong to the IncA/C, IncF, IncH, IncX and IncR incompatibility groups (4, 5).

The present study characterizes the genetic environment of the *bla*_NDM-1_ gene harbored in IncR plasmids from Enterobacter hormaechei clinical isolates involved in an outbreak in Quebec.

Three clinical isolates collected from hospitalized patients in August 2020, exhibited carbapenemase activity using the modified carbapenem inactivation method (6). These isolates were shown to harbor the *bla*_NDM_ carbapenemase gene as detected by an in-house modified multiplex Real-Time PCR protocol (7–9), and were subsequently sent to the reference laboratory of Quebec in order to investigate a putative outbreak. Pulsed-field gel electrophoresis (PFGE) analysis revealed that 2 of the 3 the clinical isolates were indistinguishable (data not shown).

In order to determine if a common plasmid was responsible for NDM dissemination, whole-genome sequencing (WGS) was performed on the isolates recovered from the 3 patients using short read (NextSeq, Illumina) and long read (MinION, Oxford Nanopore Technologies) technologies with subsequent hybrid assemblies done using Unicycler v0.4.7 (10). The antimicrobial susceptibility testing was performed using Sensititre plates (Thermo Fisher Scientific) and Etest gradient strips (bioMérieux) according to the manufacturer’s instructions. MIC interpretation was done following CLSI guidelines (6). The analysis revealed one isolate to be an *E. hormaechei* subsp. *xiangfangensis* which harbored *bla*_NDM-1_ on a 294 600 bp IncHI2/HI2A multidrug resistance plasmid. This isolate was ST171, a sequence type which has been found to be widely disseminated in the USA (11) and was not further studied here. The two related isolates (N20519 and N20520) were determined to be *E. hormaechei* subsp. *steigerwaltii* ST177 that harbored two MDR plasmids. Each harbored a 128 121 bp conjugative IncFII plasmid that interestingly, harbored *bla*_LAP-2_, a rare class A narrow spectrum enzyme initially found in an E. cloacae isolate from China (12). The *bla*_NDM-1_ genes were found on almost identical 44 kb IncR plasmids that only differed by 405 bp due to different numbers of iterons in the iteron II region. BLAST analysis against the GenBank database using the nucleotide sequence of one of these IncR plasmids, pN20519NDM, revealed 2 IncR plasmids and 8 hybrid plasmids with an IncR replicon, all of which harbored an NDM gene that shared closely related IncR regions and somewhat similar MDR regions. A BLAST Atlas of these 10 plasmids against pN20519NDM is shown in Fig. 1A. Analysis showed the presence of 3 major regions: an IncR backbone of ∼12 kb common to all, a MDR region of ∼20 kb present in 5/10 of the plasmids, which consisted of a ∼5 kb *IS26-mph*(A)*-mrx-mphR*(A)*-IS6100* azithromycin resistance unit we previously evidenced in *Shigella* (13), and a novel ∼6-13 kb complex class 1 integron region. This novel class 1 integron complex in the pN20519NDM plasmid consisted of two variable regions separated by an ISCR1 element. The class 1 integron was responsible for the acquisition of *bla*_NDM-1_ as well as other resistance genes such as *bla*_OXA-1_, *bla*_OXA-10_, *sul1*, *ant*(3′)-la, *arr-3*, c*atB3* and *aac(6′)-Ib-cr*. Plasmids with the most similar complex class 1 integrons were pCB1_SE1_NDM (IncFIB/IncFII, 171 645 bp) which harbored *dhfr1* and *aadA16* cassettes instead of *bla*_OXA-1_ and *catB3*, and pMBR_DHA-1_1C (IncR, 54 471 bp) in which the *trpF-ble*_MBL_-*bla*_NDM_-*aadA1* region downstream of ISCR1 in pN20519NDM had been replaced by a 13.6 kb region harboring *bla*_DHA-1_, shown in Fig. 1B. Interestingly, the shared regions of pMBR_DHA-1_1C (found in a Klebsiella pneumoniae isolated from a cat in Switzerland) and pN20519NDM share > 99% identity.

**FIG 1:**
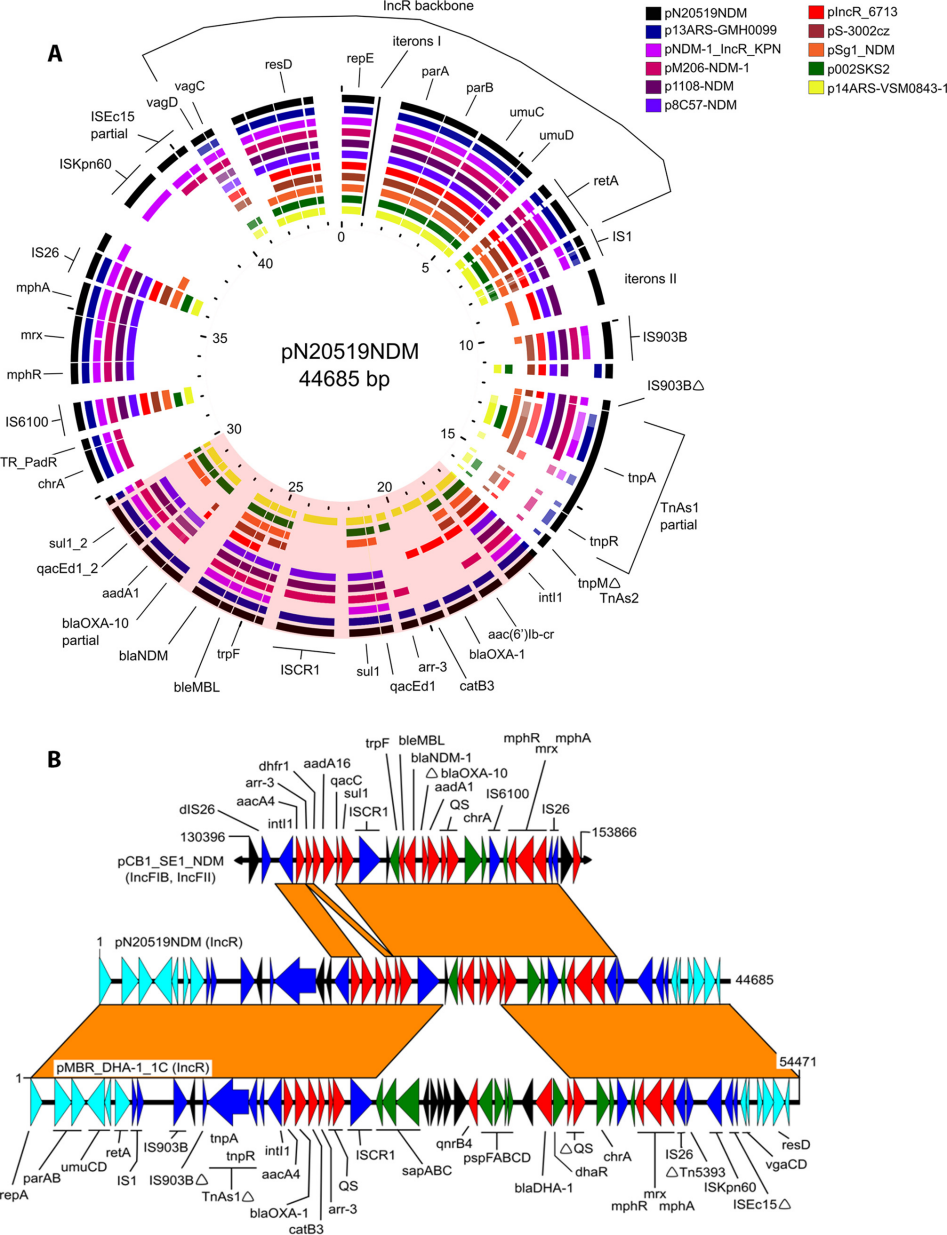
(A) BLAST Atlas comparisons of 10 IncR replicon-containing plasmids against pN20519NDM generated by the Gview Server (https://server.gview.ca). GenBank accession numbers are the following: p13ARS-GMH0099 (LR697099), pNDM-1_IncR_KPN (CABHKL010000003.1), pM206-NDM-1 (AP018830), p1108-NDM (MG825381), p8C57-NDM (MT407546), pIncR_6713 (MT415057), pS-3002cz (KJ958927), pSg1_NDM (CP011839), p002SK2 (CP025517) and, p14ARS-VSM0843-1 (LR697132). The complex class 1 integron region is shaded. The pNDM-1_IncR_KPN and pSg1-NDM are solely IncR plasmids while the others are hybrid plasmids also containing IncFIA (HI1) (p002SKS2, p14ARS_VSM0843-1, pS-3002cz), IncC and IncFIA (HI1) (p13ARS_GMH099), IncN and IncX (p1108-NDM), IncFII(K) and a partial IncQ1 (pM206-NDM-1), or IncX1 (p8C57-NDM). (B) Alignment of pN20519NDM with the plasmid or plasmid region with the most similar complex class 1 integron regions as determined by BLAST. Orange boxes indicate regions of >90% identity. The horizontal arrows indicate open reading frames and represent IncR backbone genes (light blue), mobile element-related genes (dark blue), antimicrobial resistance genes (red), other functional genes (green) and hypothetical protein genes (black). GenBank accession numbers are pMBR_DHA-1_1C (CP049955) and pCB1_SE1_NDM (MK124610). The delta symbols (Δ) indicate truncation and/or partial sequences.

The copresence of diverse and numerous mobile genetic elements such as an azithromycin resistance region linked to a complex class 1 integron demonstrates the potential of such small nonconjugative plasmids to serve as a reservoir of mobile AMR genes, as confirmed by the resistance profile it exhibited, shown in Table 1. Indeed, the strain was resistant to multiple classes of antibiotics, including carbapenems, cephalosporins, β-lactam/β-lactamase inhibitors, fluoroquinolones, aminoglycosides, sulfonamides, macrolides, and rifamycins. These sizable pools of resistance genes appear to be able to be transferred via the ability of IncR replicons to become integrated into conjugative plasmids thus forming large self-transferable multireplicon plasmids. In addition, a shared presence with a conjugative plasmid, as was the case in the isolate N20519, also enables its transfer to new recipients via conjugation.

**TABLE 1:** Antimicrobial susceptibility of *E. hormaechei* N20519 as determined by Sensititre and Etest^*a*^

Antimicrobial	MIC (µg/ml)	Interpretation^*b*^	Antibiotic class	Antibiotic resistance gene^*c*^
Sensititre^*a*^				
Aztreonam	16	R	β-lactams	*bla_OXA-10_* *bla_OXA-1_* *bla_NDM-1_*
Cefepime	>16	R		
Ceftazidime	>16	R		
Ceftriaxone	>32	R		
Ertapenem	>2	R		
Meropenem	8	R		
Ceftazidime-Avibactam	>16	R	β-lactams/β-lactamase inhibitor	
Ceftolozane-Tazobactam	>8	R		
Meropenem/Varborbactam	8	I		
Imipenem/Relebactam	8	R		
Piperacillin-Tazobactam	>64	R		
Ciprofloxacin	>2	R	Fluoroquinolones	*aac(6’)-Ib-cr*
Levofloxacin	4	R		
Doxycycline	≤4	S	Tetracyclines	
Minocycline	≤4	S		
Tigecycline	≤0.5	S		
Gentamicin	>8	R	Aminoglycosides	*aac(6’)-Ib-cr* *aadA1* *ant(3′)-la*
Tobramycin	8	I		
Amikacin	≤8	S		
Plazomicin	≤1	S		
Colistin	≤1	I	Polymyxins	
Trimethoprim/Sulfamethoxazole	>4	R	Sulfonamides	*sul1*
Etest				
Azithromycin	>256	R	Macrolides	*mphA*
Rifampicin	>32	R	Rifamycins	*arr-3*
Others				
	ND	ND	Chloramphenicol	*catB3*
	ND	ND	Bleomycin	*ble_MBL_*

a Sensititre CAN1MTSF plate (Thermo Fisher Scientific).

b Categorical interpretations based on CLSI guidelines (4).

c Antimicrobial resistance gene found in pN20519NDM as determined by Resfinder (cge.cbs.dtu.dk/services/Resfinder/).

Our results also demonstrate that the AMR genes detected by WGS correlates with the phenotypic MIC data we obtained not only for antibiotics of interest, such as the carbapenems, but also for several other classes of antibiotics not targeted for Enterobacter hormaechei. Indisputably, this powerful information supports the building knowledge surrounding antimicrobial resistance predictions in the WGS era. Interestingly, the two patients sharing an identical pulsovar and IncR plasmid, also share epidemiologic factors. The first patient who was suffering from an osteomyelitis received piperacillin-tazobactam for 6 weeks followed by a 1-week course of meropenem 2 weeks later. An NDM-positive result was obtained from a clinical sample (wound infection) during foot surgery while rectal swabs still remained negative. The other patient, who was suffering from diabetes, dyslipidemia, and heart failure, came from a long-term care residential center and exhibited an NDM-positive rectal swab 6 days after admission and was not screened for NDM upon arrival. The patient was put on piperacillin-tazobactam at admission for an infected wound. Then, he exhibited a CPE-positive result from rectal screening. Both patients were epidemiologically linked as they were treated at the same ambulatory care clinic prior to admission.

The well-established phenomenon of resistance to antibiotics is the direct consequence of our propensity to use last-resort antibiotics such as carbapenems for managing bacterial infections thus leading to an increase in the selection of carbapenem-resistant bacteria. Today, most CPEs are nosocomially acquired from indirect contacts and reminds us that the best practice in hospital settings remains the only guarantee to minimize this scourge.

The rapid identification and characterization of resistance gene environments are therefore assets and represent useful information for surveillance and patient management.

**Accession numbers.** Sequences were deposited in GenBank with the following accession numbers: Plasmids from strains N20519 and N20520 were designated pN20519NDM (Accession: NZ_MW192782.1) and pN20520NDM (Accession: NZ_MW192783.1), respectively.
